# Interventive psychodiagnosis of children through online orientation of parents in a University Clinical Practice in Brazil: an alternative for underserved populations during the COVID-19 pandemic

**DOI:** 10.1192/j.eurpsy.2022.1470

**Published:** 2022-09-01

**Authors:** C. Varanda, M. Campos

**Affiliations:** Universidade Paulista, Instituto De Ciências Humanas, Santos, Brazil

**Keywords:** psychodiagnosis add children add orientation to parents add online

## Abstract

**Introduction:**

A private university in Santos offers a free psychological service for assessing and intervening in chilhood psychological problems through a internship program which had to be delivered online due to the COVID-19 pandemic. The interns were only allowed to attend their parents online, instead of their children.

**Objectives:**

Evaluating this new online service is the aim of this work.

**Methods:**

24 parents of 34 children aged 4-10 years were attended by pairs or trios of 52 interns. The were modules made up of assessment, intervention and feedback, using different instruments such as interviews, screening questionnaires and the observation of 5-minute free play in domestic environment and of a family collage through a video recorded by parents. Feedback and intervention happened in various moments. The interns created a storybook using metaphoric narrative as a feedback tool in which a synthesis of the psychodiagnostic process and orientation was presented to the children.

**Results:**

There was progress and decrease or elimination of symptons in 19 of the 34 children. Among the children who did not improve, one of them did not present any difficulties; 7 of them had many absences and the other 7 were referred to further assessment for reasons related to the complexity of their difficulties or a probable unsuitability of the online orientation.

**Conclusions:**

The orientation was helpful for 55,89% of the children, showing to be a valid alternative for families who do not have financial resources for attending private clinics or fail to access public health services or even during social distance measures.

**Disclosure:**

No significant relationships.

